# Prospective multicenter evaluation of an easy-to-use DNA microarray for the detection of dermatophytes in clinical samples compared with conventional mycological methods

**DOI:** 10.1128/spectrum.00567-26

**Published:** 2026-08-28

**Authors:** Hélène Guegan, Geneviève Cremer, Françoise Foulet, Françoise Botterel, Arnaud Jabet, Frédérique Boquel, Alicia Moreno-Sabater, Jean-Pierre Gangneux

**Affiliations:** 1 Laboratoire de Parasitologie-Mycologie, European ECMM Excellence Center in Medical Mycology, Centre National de Référence pour les mycoses et antifongiques- Laboratoire associé Aspergilloses chroniques, CHU Rennes, Rennes, France; 2 Univ Rennes, CHU Rennes, Inserm, Irset (Institut de Recherche en Santé, Environnement et Travail), UMR-S 1085 36684, Rennes, France; 3 Laboratoire Bioclinic Madeleine, Groupe Inovie, Paris, France; 4 Département Prévention, Diagnostic, Traitement des Infections, Unité de Parasitologie-Mycologie, AP-HP, Hôpitaux universitaires Henri Mondor, Paris-Est Créteil University 378967, Créteil, France; 5 Laboratoire de Parasitologie-Mycologie, Hôpital Saint-Antoine 37117, Paris, France; 6 Laboratoire de Parasitologie-Mycologie, Hôpital de la Pitié-Salpêtrière 26933, Paris, France; 7 IPLESP (Institut Pierre Louis d’Epidémiologie et de Santé Publique), INSERM, Sorbonne Université 27063, Paris, France; 8 Sorbonne Université, Centre d'Immunologie et des Maladies Infectieuses, (CIMI-PARIS), Inserm U1135 27063, Paris, France; National Institute for Communicable Diseases, Johannesburg, Gauteng, South Africa

**Keywords:** DNA chip, PCR, dermatophyte, onychomycosis

## Abstract

Molecular methods are increasingly used for the diagnosis of superficial fungal infections, providing rapid and sensitive detection of dermatophytes. This multicenter study evaluated the diagnostic performance of the DendrisKIT (DP assay), an automated DNA microarray for dermatophyte detection, compared with conventional mycological methods. In total, 490 clinical samples from four French centers were analyzed using the DP assay, direct examination (DE), and fungal culture. After excluding invalid results, 487 samples (316 nail samples, 145 skin scrapings, 26 scalp samples) were included. Discrepant results were further investigated using an alternative commercial molecular assay. The DP assay detected dermatophytes in 367/487 samples (75.4%), outperforming DE (67.4%) and culture (63.4%). Compared with culture, the DP assay showed that the sensitivity to culture was 96.4% [94.3–98.5], while the specificity was 61.2% [54.0–68.4], reflecting additional detections rather than false positives. Among culture-negative/DP assay-positive samples, 58 were considered probable true positives, leading to an adjusted specificity of 90.8% [95%CI: 85.6–96.0]. Eleven culture-positive/DP-negative cases were observed, likely due to heterogeneous fungal distribution within the sample. The highest detection rates were achieved by combining the DP assay with DE or culture (80.9% and 77.6%, respectively). The DP assay is a reliable, easy-to-use, rapid, and high-throughput method for dermatophyte detection. When used with conventional mycological methods, it improves diagnostic yield and reduces turnaround time, but culture remains essential for identifying rare fungi causing superficial fungal infections but not included in the panel, such as non-dermatophyte molds.

**IMPORTANCE:**

Commercial multiplex assays improve dermatophyte detection in clinical samples, but their use is limited by poor standardization and accreditation constraints. DNA microarray technology expands pathogen detection and offers a promising alternative to multiplex real-time PCR. Here, we evaluated the DendrisKIT DP DNA microarray in a large multicenter cohort, showing improved diagnostic yield and shorter turnaround times, while culture remains essential for detecting rare fungi, including non-dermatophyte molds not covered by the panel.

## INTRODUCTION

Dermatomycoses are superficial fungal infections that remain a major public health concern worldwide. They are widespread, and their clinical presentation depends on the site of infection. Dermatophytes are keratinophilic fungi responsible for onychomycosis (nail infections), tinea corporis (skin infections), and tinea capitis (scalp infections). The nature of fungal species identified in human samples varies according to the geographical area and the site of sample collection. The two most prevalent dermatophytes responsible for toenail infections are *Trichophyton rubrum* and *T. interdigitale*, whereas fingernail infections are predominantly caused by yeasts such as *Candida albicans* (1). Toenail infections may also, less frequently, be caused by non-dermatophyte molds (NDMs), such as *Fusarium* spp., *Neoscytalidium* spp., and *Aspergillus* spp., which account for 6.9% of onychomycoses worldwide, with significant geographical disparities (1).

The current epidemiology of superficial fungal infections is also marked by the widespread emergence of terbinafine-resistant dermatophyte strains, such as *T. indotineae*, which are associated with high rates of treatment failure and require proper diagnosis (2, 3).

Accurate identification of the dermatophyte genus—distinguishing between *Trichophyton* and *Microsporum* in cases of tinea capitis, in particular—is crucial for the selection of an effective antifungal therapy. In several countries, guidelines for treatment have recently shifted from griseofulvin to newer agents, such as terbinafine or itraconazole, depending on the species involved (4). This change in therapeutic approach underscores the importance of precise diagnosis in the management of tinea capitis (5, 6).

Conventional laboratory methods for diagnosing dermatomycosis include both microscopy-based direct examination (DE) and the culture of fungi from skin scrapings, nail clippings, or plucked hair. DE involves placing a sample between a slide and coverslip with a brightening agent to facilitate the visualization of fungal hyphae. However, this approach is subject to several limitations, including a lack of sensitivity, resulting in 5% to 15% false-negative results (7). It is also dependent on the microscopy skills of the operator, and there is a potential for confusion between dermatophytes, molds, and yeast hyphae, particularly in non-specialized laboratories. Fungal culture—the current reference standard method—can identify viable fungi, but the turnaround time is long, as it typically requires at least two weeks of incubation on Sabouraud medium to obtain a result. Accurate identification to species level may require additional subcultures on nutrient-poor media to promote fructification.

Molecular diagnostic tools, such as PCR-based methods in particular, have been shown to enhance fungal detection, and to be both sensitive and rapid for dermatophyte detection (8). These tools may also enhance the detection of dermatophytes in specimens obtained under suboptimal sampling conditions due to poor training of the person taking the sample, for example, a limitation that remains common in routine diagnostic practice. Commercial multiplex assays are increasingly being reported to increase the rate of dermatophyte detection in clinical samples (9–15), but their use remains limited, and a lack of standardization creates challenges for compliance with accreditation requirements in clinical laboratories.

Furthermore, the inherent limitations of multiplexing capacity limit the number of fungal species targeted by these assays to the most prevalent pathogens.

In this context, DNA microarray technology broadens the spectrum of pathogens that can be detected, thereby constituting a potential alternative to multiplex real-time PCR (16). The complexity of earlier platforms hindered their clinical implementation, but the recent development of user-friendly microarrays specifically designed for dermatophyte detection warrants a new evaluation in clinical settings (17, 18).

In this study, we evaluated the performance of the DendrisKIT DP (DP assay) in a large, multicenter cohort study of clinical samples. The assay tested detects 12 fungal targets associated with superficial fungal infections. It was compared with conventional mycological methods of diagnosis.

## MATERIALS AND METHODS

### Population and samples

In total, 490 nail clippings, skin scrapings, and hair samples from 407 patients were included in this study prospectively, from January 2023 to February 2024, at four French mycology laboratories located at the Rennes, Henri Mondor, and Saint-Antoine University Hospitals, and the Bioclinic Madeleine Laboratory. All samples from patients with suspected superficial fungal infections (mostly referred by a general practitioner or a dermatologist) were included, provided that the specimen quantity was sufficient for both conventional mycology and molecular testing. Patients were asked to leave a treatment-free window before sampling if they had already begun antifungal treatment: 3 months for systemic antifungal therapy and antifungal nail lacquer, and 2 weeks for topical antifungal creams. However, ongoing antifungal treatment was not considered an exclusion criterion, given the current emergence of terbinafine-resistant strains.

Data regarding prior antifungal treatment were not systematically analyzed for all patients. Treatment history was only reviewed in cases with discordant results, defined as a positive DP assay and a negative dermatophyte culture. Samples were subjected to mycological culture as part of the routine workflow for dermatophyte detection, and any remaining samples were stored at room temperature until molecular testing. According to French Public Health Law, protocols of this type do not require approval from an ethics committee and are exempt from the requirement for formal informed consent (19). Samples were rendered anonymous before processing and data analysis.

### Standard mycological diagnosis

Routine sample processing involved a direct examination of KOH-brightened samples and mycological cultures on Sabouraud medium and Sabouraud medium supplemented with cycloheximide. At one of the centers, two additional media were used: a cycloheximide-enriched brain heart infusion medium to increase the growth of slow-growing dermatophytes, such as *T. verrucosum,* and a low-glucose medium to favor dermatophyte sporulation. All culture media were incubated at 27°C ± 2°C for 3 to 4 weeks, depending on the laboratory. Yeast strains were identified by MALDI-ToF mass spectrometry with a MALDI Biotyper (Bruker Daltonics) and the manufacturer’s database. Isolates of filamentous fungi were identified according to macroscopic and microscopic morphological criteria at all centers. Some strains with poor sporulation were subcultured on potato carrot agar to promote fructification. Dermatophytes for which identification was uncertain were analyzed by MALDI-ToF mass spectrometry with a MALDI Biotyper (Bruker Daltonics), the manufacturer’s database, and the MSI-2 database (20, 21), at all but one of the centers.

### Sample pretreatment and DNA extraction

Nail, skin, and hair fragments (10 to 60 mg) were transferred to 1.5-mL tubes and suspended in 400 µL DLB Dendris buffer (Dendris, Labège, France). We added 10 µL proteinase K (DPK, Dendris) and incubated the samples for 3 h at 56°C, as recommended by the manufacturer. Of note, bead beating was omitted for nail samples because the specimens were collected as a fine powder. A preliminary experiment indicated that bead beating increased the release of PCR inhibitors without providing a consistent improvement in DNA recovery. Because the amount of DNA recovered is dependent on both the nature of the specimen and the quality of sampling, the input material was standardized to 10–60 mg of specimen rather than by the amount of extracted DNA. Samples were stored at −20°C until DNA extraction. Before loading on the extraction machine, tubes were vortexed and centrifuged for 1 min at 20,800 × *g* and 20°C. After PK lysis, DNA was extracted from 200-µL lysate on a MICROLAB STARlet device (Hamilton Company, Reno, NV, USA) with STARMag 96 Universal Cartridge reagent (Seegene Inc., Seoul, South Korea). DNA samples were stored frozen at −20°C until testing.

### PCR amplification

Dermatophyte detection was performed with the DendrisKIT DP assay (DP assay, CE-IVD, Dendris, Labège, France), which simultaneously amplifies 12 targets: *Trichophyton* spp., *T. rubrum, T. mentagrophytes/interdigitale, T. tonsurans, T. verrucosum, T. soudanense, T. benhamiae*, *T. violaceum, Microsporum* spp., *Nannizia gypsea*, *Epidermophyton floccosum,* and *Candida albicans*. We would expect the emerging species *T. indotineae* to be detected as *T. mentagrophytes/interdigitale*, without specific identification. The first step was a full ITS-targeted real-time PCR conducted in a 96-well plate on a CFX96 device (Biorad, Marne-la-Coquette, France) with the reagent provided in the DP assay (up to 94 clinical samples/plate). Sample quality was also checked by a concomitant PCR targeting the human 18S gene as an internal control (IC). In accordance with the manufacturer’s recommendations, any signal below 37 quantification cycles (Cq) was considered positive. A sample was not evaluable if PCR tests were negative for both 18S and ITS. By contrast, any sample with a valid 18S-based IC and a negative ITS PCR required confirmation on the DendrisCHIP DP (a sample can be ITS PCR-negative but hybridization-positive).

### DendrisCHIP DP processing

PCR products were loaded onto an automated system (DendriSTATION, Dendris), which performed the entire hybridization procedure for up to 64 samples per run, including PCR product purification, hybridization on DNA Chips (DendrisCHIP, Dendris), conjugation, elution, and detection of hybridization on the chips. The chips were then read with a dedicated scanner (DendriSCAN, Dendris), and the results were analyzed with software supplied by the manufacturer (DendriSOFT), including the validation of hybridization controls.

The result was delivered in about 4 h as a prediction of pathogen presence or absence using a supervised multilabel machine-learning algorithm based on the HOMER classification method combined with random forest learning, trained on a large fungal strains collection. The database could potentially be expanded to include additional clinical hybridization profiles (17). No updates to the database were made during the study, and all samples were analyzed using the same reference database.

### DermaGenius dermatophyte assay

DNA samples for which identification was discordant between DP assay and culture, and samples with an isolated positive DP assay signal, were analyzed with the DermaGenius real-time multiplex PCR for confirmation (PathoNostics, the Netherlands). Amplification and melting curve analysis were performed on a LightCycler 480 II (Roche) according to the manufacturer’s instructions. Data were analyzed by the second-derivative method in the Tm-calling mode of LC480 software.

### Statistical analysis

Statistical analysis was performed with GraphPad Prism software v.8.0.2 (San Diego, CA). The crude concordance between culture and PCR for dermatophyte detection (*C. albicans* not included) was analyzed by calculating Cohen’s kappa coefficient. The sensitivity and specificity of the DP assay for dermatophyte detection were assessed relative to conventional mycological tools: positive samples were defined as those with a positive DE and/or positive fungal culture; negative samples were defined as those with a negative DE and negative fungal culture. The 95% confidence intervals [95% CI] for proportions were calculated using the normal approximation to the binomial distribution.

## RESULTS

### Sample characteristics and mycological diagnosis

In total, 490 samples received for routine mycological diagnosis were subjected to the DP assay. Three of these samples were excluded due to an invalid 18S PCR control (*n* = 2) or invalid hybridization control (*n* = 1). Thus, a total of 487 samples were analyzed from 404 patients: 316 samples, 145 skin scrapings, and 26 scalp samples. Three hundred and thirty-three patients (82.4%) had only one sample analyzed, 60 (14.9%) had two samples included in the study, and 11 (2.7%) had three or more samples from different body sites included in the study.

In total, 360 samples (73.9%) yielded a positive direct examination (DE) result showing fungal structures (nonspecific of dermatophytes), or a positive dermatophyte culture, and dermatophytes were isolated from 309 of these samples (85.8%) in culture (Table 1).

**TABLE 1:** Standard mycology results for dermatophyte detection (direct examination, mycological culture)

	Positive DE^*a*^^**,*b***^ *n* (%)	Positive dermatophyte culture *n* (%)	Positive DE^*a*^^,*b*^ and/or dermatophyte culture *n* (%)
All samples *N* = 487	328 (67.4)	309 (63.4)	360 (73.9)
Nails *N* = 316	223 (70.6)	197 (62.3)	240 (75.9)
Foot *n* = 309	221 (71.5)	195 (63.1)	237 (76.7)
Hand *n* = 7	2 (28.6)	2 (28.6)	3 (42.9)
Skin *N* = 145	93 (64.1)	97 (66.9)	105 (72.4)
Foot/hand *n* = 122	81 (66.4)	81 (66.4)	89 (73.0)
Other sites *n* = 23	12 (52.2)	16 (69.6)	16 (69.6)
Scalp (skin scrapings ± hair) *N* = 26	12 (46.2)	15 (57.7)	15 (57.7)

^*a*^ DE, direct examination.

^*b*^ Hyphae from filamentous fungi or spores, nonspecific of dermatophytes.

*Trichophyton rubrum* was by far the most prevalent species cultured, detected in 250/309 samples (80.9%), followed by *Trichophyton mentagrophytes/interdigitale* in 39/309 (12.6%). Scalp samples yielded *Microsporum audouinii* (7/15, 46.7%), *Trichophyton tonsurans* (7/15, 46.7%), and *Trichophyton violaceum/soudanense* (2/15, 13.3%).

In addition to dermatophytes, 23 samples were positive for non-dermatophyte molds, including *Fusarium* spp. (*n* = 9), *Aspergillus* spp. (*n* = 8), *Acremonium* spp. (*n* = 4), *Alternaria* spp. (*n* = 1), and *Chaetomium* spp. (*n* = 1). Thirteen of these 23 samples yielded positive results on direct microscopic examination (5 *Fusarium*, 5 *Aspergillus*, 2 *Acremonium*, and 1 *Alternaria*), suggesting a potential diagnosis of non-dermatophyte mold onychomycosis although diagnostic criteria for NDM onychomycosis were not applied here. *Candida albicans* was isolated from seven samples (six nail clippings and one skin scraping), five of which were positive on direct examination. Two samples were found to be co-infected with *T. rubrum*.

### Performance of the DP assay relative to direct examination and culture for the detection of dermatophytes

The DP assay detected dermatophytes in 367 of the 487 samples analyzed (75.4%), whereas dermatophytes were detected in only 309 (63.4%) samples by culture (Table 2). The global concordance between culture and the DP assay for dermatophyte detection was 83.6% [95%CI: 80.3–86.9] (407/487) with a Cohen’s Kappa coefficient of 0.620 ± 0.037 [0.547–0.692]. The sensitivity of the DP assay relative to culture was 96.4% [95%CI: 94.3–98.5] (298/309), ranging from 87.5% [95%CI: 76.0–99.0] for DE-negative samples to 97.5% [95%CI: 85.7–99.3] for DE-positive samples (Table 2). The sensitivity of the DP assay varied slightly with sample type, from 86.7% [95%CI: 69.5–100] for scalp samples to 97% [95%CI: 94.6–99.4] for nail samples, relative to culture as the reference (Table 3). Relative to the combination of DE and culture, the sensitivity of the DP assay on nail samples was 90% [95%CI: 86.2–93.8], due to the broader range of fungi detected by DE than with the DP assay.

**TABLE 2:** Results of the DP assay according to standard mycology results for dermatophyte detection

	Number of samples (%)						
	All samples	Positive culture for dermatophytes			Negative culture for dermatophytes		
	***N*** = 487	All ***N*** = 309	DE-positive N = ***277***	DE-negative *N* = 32	All ***N*** = 178	DE-positive *N* = 51	DE-negative *N* = 127
DP assay negative for dermatophytes	120 (24.6)	11 (3.6)	7 (2.5)	4 (12.5)	109 (61.2)	20 (39.2)	89 (70.1)
DP assay positive for dermatophytes	367 (75.4)	298 (96.4)	270 (97.5)	28 (87.5)	69 (38.8)	31 (60.8)	38 (30.0)
*T. rubrum*	271 (55.6)	237 (76.7)	222 (80.1)	15 (46.9)	34 (19.1)	16 (31.4)	18 (14.2)
*T. mentagrophytes/ interdigitale*	47 (9.7)	35 (11.3)	28 (10.1)	7 (21.9)	12 (6.7)	6 (11.8)	6 (4.7)
*T. tonsurans*	3 (0.6)	3 (1.0)	3 (1.1)	0	0	0	0
*T. soudanense*	1 (0.2)	1 (0.3)	1 (0.4)	0	0	0	0
*T. benhamiae*	1 (0.2)	1 (0.3)	0	1 (3.1)	0	0	0
*T. verrucosum*	1 (0.2)	1 (0.3)	1 (0.4)	0	0	0	0
*Trichophyton* spp.^a^	34 (7.0)	12 (3.9)	7 (2.5)	5 (15.6)	22 (12.4)	8 (15.7)	14 (11.0)
*Microsporum* spp.	6 (1.2)	6 (1.9)	6 (2.2)	0	0	0	0
*E. floccosum*	3 (0.6)	2 (0.6)	2 (0.7)	0	1 (0.6)	1 (2.0)	0

^a^ The *Trichophyton* spp. designation is defined by the manufacturer and may refer to any *Trichophyton* species, including those for which species-specific probes are available. This result may arise from partial amplicon hybridization on the microarray, leading to an incomplete hybridization pattern that cannot be resolved to the species level by the AI-based algorithm.

**TABLE 3:** Sensitivity, specificity, PPV^*a*^, NPV^*a*^, and adjusted specificity of the DP assay relative to different reference standards

	Relative sensitivity *n/N* (%, [95% CI])		Relative specificity^*b*^ *n/N* (%, [95% CI])		PPV (%, [95% CI])	NPV (%, [95% CI])	Adjusted specificity^*c*^ n/N (%, [95% CI])	
**Reference: mycological culture**								
All	298/309	96.4 [94.3–98.5]	109/178	61.2 [54.0–68.4]	81.2 [77.2–85.2]	90.8 [85.6–96.0]	109/120	90.8 [85.7–96.0]
Nail clippings	191/197	97.0 [94.6–99.4]	68/119	57.1 [48.2–66.0]	78.9 [73.8–84.0]	91.9 [85.7–98.1]	68/73	93.1 [87.4–98.9]
Skin scrapings	94/97	96.9 [93.5–100]	32/48	66.7 [53.4–80.0]	85.5 [78.9–92.1]	91.4 [82.1–100]	32/37	86.5 [75.5–97.5]
Scalp samples	13/15	86.7 [69.5–100]	9/11	81.8 [59.0–100]	86.7 [69.5–100]	81.8 [59.0–100]	9/10	90.0 [71.4–100]
**Reference: DE + mycological culture**								
All	329/360	91.4 [88.5–94.3]	89/127	70.1 [62.1–78.1]	89.6 [86.5–92.7]	74.2 [66.4–82.0]		
Nail clippings	216/240	90.0 [86.2–93.8]	50/76	65.8 [55.1–76.5]	89.3 [85.4–93.2]	67.6 [56.9–78.3]		
Skin scrapings	100/105	95.2 [91.1–99.3]	30/40	75.0 [61.6–88.4]	90.9 [85.5–96.3]	85.7 [74.1–97.3]		
Scalp samples	13/15	86.7 [69.5–100]	9/11	81.8 [59.0–100]	86.7 [69.5–100]	81.8 [59.0–100]		

^*a*^ PPV, positive predictive value; NPV, negative predictive value.

^*b*^ Relative specificity was calculated as the number of samples with a negative DP assay over the number of dermatophyte-negative culture samples (culture as the reference) or over the number of samples negative for both DE and dermatophyte culture (DE + culture as the reference).

^*c*^ Adjusted specificity was calculated after reclassifying 58 culture-negative/DP assay-positive samples as probable true positives based on DE, the presence of another positive sample from the same patient, and/or positive DermaGenius PCR results.

The 11 false-negative DP assay results (identified by comparison to culture) included seven for which DE was positive (Table 2). These samples consisted of six nail clippings and three skin scrapings positive for *T. rubrum* (*n* = 8) and *T. mentagrophytes/interdigitale* (*n* = 1) on culture and two scalp samples positive for *M. audouinii* and *T. violaceum/soudanense,* respectively, by culture. No PCR inhibitor was detected in any of these samples.

The DP assay was positive for *C. albicans* for 2/7 samples with positive *C. albicans* culture and for another six samples testing negative by both DE and culture.

The relative specificity of the DP assay against culture appeared low, at 61.2% [95%CI: 54.0–68.4] (109/178) for all sample types, due to the 69 additional culture-negative samples testing positive in the DP assay (Tables 2 and 3). These 69 samples consisted of 51 nail specimens (73.9%), 16 skin scrapings (23.2%), and two scalp samples (2.9%) (Table 2). Half these samples tested positive for the *T. rubrum* target (34/69, 49.3%), whereas 22 (31.9%) were positive for *Trichophyton* spp., 12 (17.4%) for *T. mentagrophytes/T. interdigitale*, and 1 (1.4%) for *E. floccosum.* Using culture as the reference standard, the positive predictive value (PPV) and negative predictive value (NPV) of the DP assay were 81.2% [95%CI: 77.2–85.2] and 90.8% [95%CI: 85.6–96.0], respectively, Table 3. When DE and culture were combined as the reference standard, the PPV and NPV were 89.6% [95%CI: 86.5–92.7] and 74.2% [95%CI: 66.4–82.0], respectively.

### Analysis of discrepant results for negative fungal culture and positive DP assay samples

**Fig 1:**
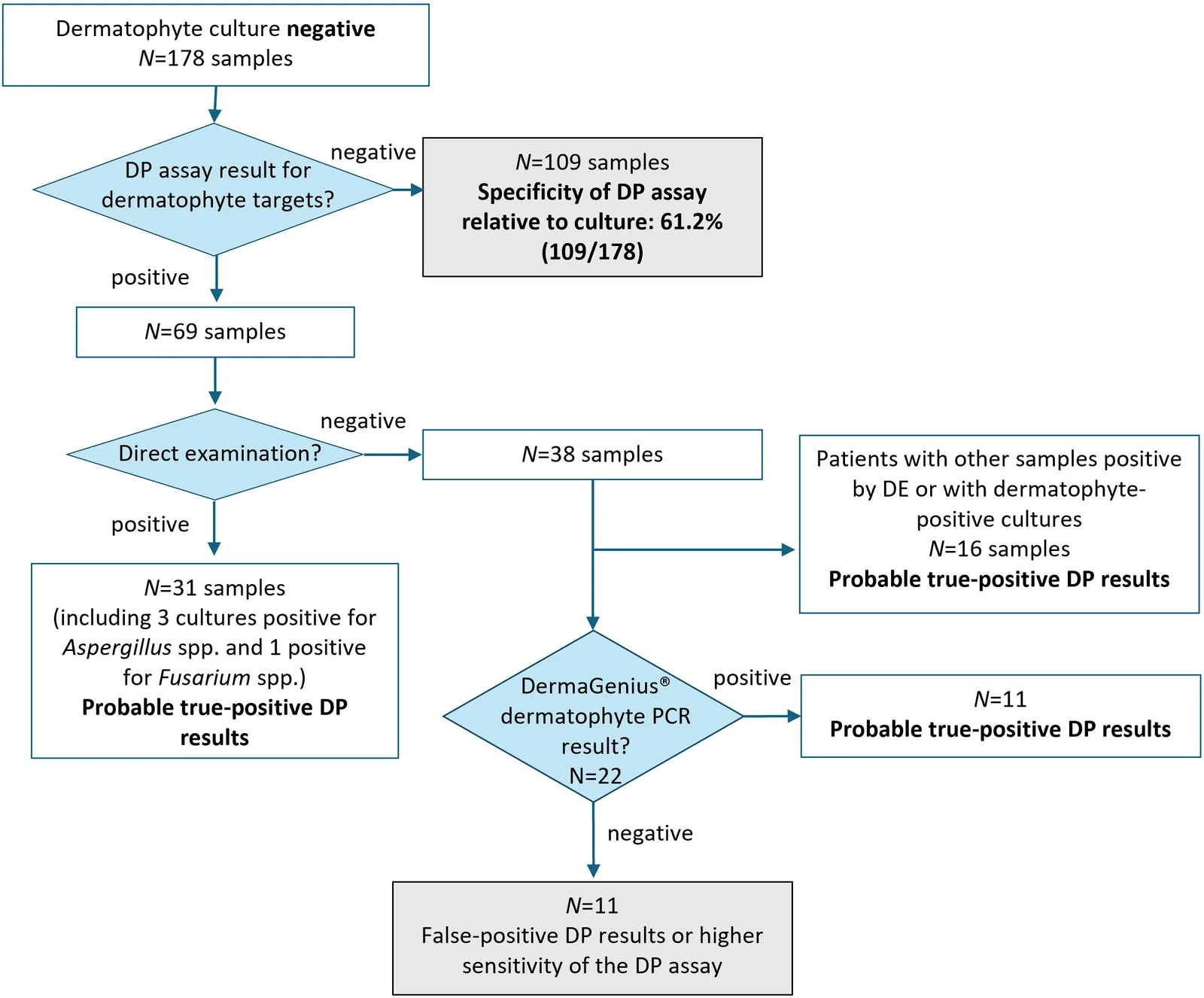
Investigation of fungal culture-negative and DP-positive samples.

A detailed analysis of dermatophyte detection by the DP assay in the 69 culture-negative samples was then performed to assess whether the results obtained were true or false positives (Fig. 1).

Thirty-one of the 69 samples (44.9%) were positive on DE, suggesting that these DP assay-positive samples were truly positive for dermatophytes. For four of these DP-positive samples, *Aspergillus* spp. (*n* = 3) or *Fusarium* spp. (*n* = 1) also grew in culture, suggesting the possibility of co-infections with molds and dermatophytes in these cases. Sixteen of the other 38 samples were collected from patients who had provided other samples displaying evidence of fungal/dermatophyte infection. The other 22 DP assay-positive samples in which *T. rubrum* (*n* = 9), *T. mentagrophytes/interdigitale* (*n* = 2), or *Trichophyton* spp. (*n* = 11) was detected were subjected to testing with the DermaGenius PCR, and 11 were confirmed to be positive for dermatophyte targets. No other evidence for dermatophyte infection was found in the remaining 11 DP assay-positive samples, all but one of which were positive for the target *Trichophyton* spp.

To assess whether prior antifungal treatment may have contributed to the 69 samples with discordant results (negative culture for dermatophytes but positive DP assay), previous antifungal exposure was investigated. Among the 62 samples for which treatment data were available, 10 (16.1%) were collected from patients who did not comply with the recommended therapeutic washout period (i.e., no treatment interruption or only a short discontinuation before sampling). Reported treatments included systemic terbinafine (*n* = 4), itraconazole (*n* = 1), and antifungal nail lacquer (*n* = 5).

Among these 10 samples, five showed positive DE results despite negative cultures, suggesting that prior antifungal treatment may have inhibited fungal growth and thereby sterilized the culture while fungal elements remained detectable by microscopy and molecular testing.

In total, 58 of the 69 culture-negative/DP assay-positive samples were considered probable true positives, which allowed the calculation of an adjusted specificity of 90.8% [95%CI: 85.6–96.0] (109/120), taking into account the additional criteria described above and detailed in Fig. 1 and Table 3.

### Analysis of identifications discrepant between fungal culture and DP assay

Six samples yielded discordant dermatophyte identification results with the DP assay and mycological culture. These samples included two identifications of *T. rubrum* and one of *T. mentagrophytes/interdigitale* in culture that yielded positive results for the *T. mentagrophytes/interdigitale* and *T. rubrum* targets, respectively. The corresponding DNA samples were amplified with the DermaGenius PCR, confirming the results of the DP assay, consistent with the hypothesis of misidentification by microscopy. For the other three samples, *T. tonsurans* grew in culture, and positive results were obtained for the *T. mentagrophytes/interdigitale* target in the DP assay. The DermaGenius PCR was positive for *T. tonsurans* for these three samples, indicating that the DP assay was less reliable than microscopy for the identification of these samples. Genus-level identification (i.e., *Trichophyton* spp.) was possible for 12 samples, yielding positive results on culture for *T. mentagrophytes/interdigitale* (*n* = 7), *T. rubrum* (*n* = 4), and *T. tonsurans* (*n* = 1). These samples had a low fungal load as the DP assay ITS PCR was negative for 4 of the 12 samples, and the other eight were ITS PCR-positive, with a median Cq value of 32.5 [28.7–35.1].

The DP assay yielded the highest overall positivity rate for dermatophyte detection (75.4%), outperforming both DE (67.4%) and mycological culture (63.4%) (Table 4). Combining the techniques increased diagnostic yield substantially: the combination of DE and DP assay yielded a positivity rate of 80.9%, whereas the combination of culture with the DP assay yielded a positivity rate of 77.6%. Nail clippings were the sample type with the highest detection rates, achieved with a combination of DE and the DP assay (83.9%). Positivity rates were slightly lower for skin scrapings, peaking at 77.9% with both combined methods. Scalp samples yielded the lowest positivity rates overall, with a maximum positivity rate of 65.4% achieved with a combination of culture and the DP assay.

**TABLE 4:** Summary of positivity rates for direct examination, mycological culture, and the DP assay, alone and in combination, for dermatophyte detection (*N* = 487)

Technique		Number of positive samples (%, [95%CI])				
		All samples *N* = 487	Nail clippings *N* = 316	Skin scrapings *N* = 145	Scalp samples *N* = 26	
Direct examination^a^		328 (67.4, [63.2–71.6])	223 (70.6, [65.6–75.6])	93 (64.1, [56.3–71.9])	12 (46.2, [27.0–65.4])	
Mycological culture for dermatophytes		309 (63.4, [59.1–67.7])	197 (62.3, [57.0–67.6])	97 (66.9, [59.2–74.6])	15 (57.7, [38.7–76.7])	
DP assay		367 (75.4, [71.6–79.2])	242 (76.6, [71.9-81.3])	110 (75.9, [68.9-82.9])	15 (57.7, [38.7–76.4])	
Direct examination and DP assay combined		394 (80.9, [77.4–84.4])	265 (83.9, [79.8–88.0])	113 (77.9, [71.1–84.7])	16 (61.5, [42.8–80.2])	
Culture and DP assay combined		378 (77.6, [73.9–81.3])	248 (78.5, [74.0–83.0])	113 (77.9, [71.1–84.7])	17 (65.4, [47.1–83.7])	

^a^ Not specific for dermatophyte detection: visualization of fungal forms without distinguishing spores/mold hyphae/dermatophyte hyphae.

## DISCUSSION

The combination of microscopy and fungal culture for the diagnosis of dermatomycosis is time-consuming, requires particular skills, and the result takes several weeks to obtain, potentially leading to incorrect treatment during the interim. Molecular tools for diagnosing superficial fungal infections are rapidly spreading and provide a rapid, sensitive detection of most of the dermatophytes implicated in human infections without the need for specialist microscopy skills. Growing numbers of commercial real-time multiplex PCRs targeting dermatophytes are being evaluated (12, 13, 22, 23), but very few data are available concerning the clinical performances of innovative and high-throughput DNA microarrays. In this multicenter study, we assessed the performance of the DendrisKIT DP assay (DP assay), an automated easy-to-use DNA chip-based method, relative to the standard mycological procedure in the largest series ever published. For 487 clinical samples from four French centers, the DP assay had a high relative sensitivity of 96.4% with culture as the reference, detecting 69 additional samples that were missed by culture, thereby increasing the global rate of dermatophyte detection from 63 to 75%. Most of the additional detections were considered probable true positives as the samples were positive on DE (with no growth with non-dermatophyte fungi), patient samples provided independent laboratory-based evidence of dermatophyte infection, or infection was confirmed by a commercially available real-time PCR assay, resulting in an adjusted specificity of 90.8%. Interestingly, 16% of the samples that were culture-negative but DP assay-positive were obtained from patients who had recently received or were on ongoing antifungal treatment that was either not discontinued or had been stopped for less than 3 months before sampling, despite our recommendation to discontinue treatment. Molecular assays may therefore be valuable for confirming the diagnosis when antifungal therapy has already been initiated and culture results are negative (22). One limitation of this study was linked to the partial data collection about the antifungal history that may have had an impact on the performance of the tests evaluated. Nevertheless, our findings underscore the added value of molecular diagnostics over culture for initial diagnosis, as DNA-based assays can detect fungal nucleic acids even when viable organisms are no longer present. Conversely, this broad sensitivity may limit the utility of these assays for post-treatment follow-up, especially in tinea capitis cases, as residual fungal DNA of no clinical relevance may still be detected; clinical examination therefore remains the reference method for assessing cure (24). Another limitation is that 17% of the patients contributed multiple samples. Although this reflects routine clinical practice, it may have introduced a degree of non-independence among observations.

By contrast, the 11 samples negative for the DP assay but positive on culture (and DE for 7/11) may reflect a heterogeneous distribution of fungal structures within the samples. This raises the issue of the importance of appropriate sample collection to optimize fungal detection. Another possible explanation is fungal polymorphism, which would have affected primer and probe binding, but fungal isolates were not sequenced.

A recently published evaluation of the DP assay reported lower diagnostic performances, including sensitivity and specificity values of 83.9% and 88.9%, respectively, for a small panel of 85 clinical samples (25). One possible explanation for these discrepancies between this previous study and our results may be differences in sample preparation—particularly the amount of material processed—and in the method used for DNA extraction method.

Another DNA microarray assay, EUROArray Dermatomycosis, has been evaluated for routine diagnostic use. It can detect an extended panel of fungal targets comprising 23 dermatophyte species, three yeasts, and three non-dermatophyte molds (18, 26). In a study including 120 mixed clinical specimens—encompassing nail, skin, and scalp samples—this assay had a sensitivity of 78.6% and a specificity of 91.7% relative to the conventional reference method (direct examination plus culture) (26). In another series of 100 nail samples, the reported sensitivity and specificity were 72.3% and 71.7%, respectively, relative to the same reference methods (18). Consistent with our findings, this study also highlighted the added diagnostic value of combining the DNA microarray with direct examination, achieving a sensitivity of 93.6%, with no loss of specificity.

The main advantage of DNA microarray approaches of this type is their broad detection spectrum, making it possible to identify more than 10 different fungal targets within a single reaction well, whereas conventional multiplex PCR assays typically detect only four to five species. Beyond the potential for species-level misidentification, including an inability to differentiate *T. indotineae* from *T. mentagrophytes* complex or to distinguish between *M. canis* and *M. audouinii,* this microarray approach is limited by its restricted target panel, which is necessarily less broad than that of fungal culture. This limitation should be taken into consideration when integrating this method into routine diagnostic workflows and should be considered carefully, particularly for superficial specimens. The detection of potential non-dermatophyte fungi associated with onychomycosis should also be preserved, especially molds, such as *Fusarium*, *Aspergillus*, *Acremonium*, and *Neoscytalidium* spp., the last of these genera being highly endemic in tropical regions (1, 27–29). The machine-learning algorithm used for hybridization profile analysis could be further refined in the future to improve identification performance. However, any such update would require validation by the manufacturer, followed by independent clinical re-validation before implementation in routine practice.

In conclusion, the implementation of DNA microarray assays within the diagnostic workflow provides a valuable screening approach, improving both diagnostic sensitivity and turnaround time. The use of these microarrays should be tailored to local fungal epidemiology and systematically combined with conventional mycological methods, which remain the reference standard for identifying non-dermatophyte molds.

## Data Availability

The data sets used during the current study are available from the corresponding author on reasonable request.
